# A New Species of *Argophyllum* (Argophyllaceae) with Notes on the Species from New Caledonia and Nickel Hyperaccumulation

**DOI:** 10.3390/plants10040701

**Published:** 2021-04-05

**Authors:** Yohan Pillon, Vanessa Hequet

**Affiliations:** 1 LSTM, IRD, INRAE, CIRAD, Institut Agro, Univ. Montpellier, 34398 Montpellier, France; 2 AMAP, IRD, Herbier NOU, 98848 Nouméa, New Caledonia; vanessa.hequet@ird.fr

**Keywords:** island, metal hyperaccumulation, Pacific, serpentine, systematics, ultramafic

## Abstract

The taxonomy of *Argophyllum* (Argophyllaceae) in New Caledonia is reviewed here. All names validly published in *Argophyllum* in this archipelago are discussed and lectotypified when necessary. A new species is described, *Argophyllum riparium* (The LSID for the name *Argophyllum riparium* is: 77216335-1) Pillon and Hequet sp. nov. *Argophyllum grunowii* and *A. ellipticum* are both species complexes in which several species previously recognized are included here as well. Seven species are recognized in New Caledonia: *A. brevipetalum*, *A. ellipticum*, *A. grunowii*, *A. montanum*, *A. nitidum*, *A. riparium* and *A. vernicosum*, all endemic. Leaf nickel content of *A. riparium* can exceed 1000 μg·g^−1^, which makes this species a nickel hyperaccumulator. Measurements with a handheld X-Ray Fluorescence (XRF) spectrometer confirmed that this was also the case for all other species from New Caledonia, except *A. nitidum*. An identification key of New Caledonian species is provided.

## 1. Introduction

*Argophyllum* J.R.Forst. and G.Forst. [1] (p. 29) is a genus of shrubs and small trees that has traditionally been placed in Saxifragaceae, or sometimes in Escalloniaceae or Grossulariaceae. Molecular phylogenetic studies [2] have indicated affinities with the genus *Corokia* A. Cunn. [3] (p. 248) and the two genera form the family Argophyllaceae [4] in the order Asterales [5] (APG I and subsequent others). Argophyllaceae are closely related to two other small families [2,6,7]: Phellinaceae, with a single genus of ten species all endemic to New Caledonia [8,9,10], and Alseuosmiaceae, with four to five genera and ten species from eastern Australia, New Guinea, New Zealand and New Caledonia [11,12,13]. The exact relationships between Argophyllaceae, Phellinaceae and Alseuosmiaceae, which form the “APA clade” or Alseuosmiineae Shipunov [14] (p. 63), are still not clear [15].

The genus *Argophyllum* was described by Johann and Georg Forster, two German botanists who travelled with James Cook during his second voyage around the world. They collected material in New Caledonia that they subsequently described as *Argophyllum nitidum* J. R. Forst. and G. Forst. [1] (p. 29). A second species, *A. ellipticum* Labill. [16] (p. 39), was collected and described by Labillardière, a member of Bruni d’Entrecasteaux’s voyage in search of the La Pérouse expedition. The genus was last revised in its entirety by Zemann [17], who recognized ten species in Australia and New Caledonia. Leaf cuticle attributed to *Argophyllum* have been described from the Miocene of New Zealand [18]. The species in Australia have recently been revised with a total of eleven endemic species [19,20]. Guillaumin [21] revised the New Caledonia species as part of his revision of New Caledonian “Saxifragaceae” and recognised seven species, to which he subsequently added three new species [22]. Here, we provide a taxonomic update of New Caledonian *Argophyllum* to complete work on the genera from New Caledonia previously included in “Saxifragaceae” [23,24].

High nickel content was previously reported in the leaves of New Caledonian *Argophyllum* [25,26]. However, because the taxonomy of the genus was not clear at that time, there was uncertainty regarding the number and the names of the species that can actually be considered as nickel hyperaccumulators [27,28]. Here, we provide new analyses of herbarium specimens [29] and field-collected material to update the list of nickel hyperaccumulators in the genus *Argophyllum*.

## 2. Materials and Methods

Measurements, shapes and colours of the different organs are based on the examination of herbarium material and field observations. All herbarium specimens of *Argophyllum* present at NOU and P were examined (for herbarium acronyms: [30]). Additional type material was viewed online.

Nickel content in herbarium specimens was measured with a handheld X-Ray Fluorescence (XRF) spectrometer [28,31]. For each species, we selected specimens to represent their morphological, geographical and ecological range. For a putative new species, one leaf from five individuals was collected in the field, dried, ground to powder, digested in HNO_3_/H_2_O_2_ and analysed by Microwave Plasma-Atomic Emission Spectrometer (MP-AES) [32].

## 3. Results

Examination of herbarium specimens and observations in the field suggests that seven endemic species should be recognized in the genus. Some material resembling *A. vernicosum* Däniker [33] (p. 165) but with larger leaves, thicker twigs, sparser branching, sepals with a different shape, and a riparian ecology are placed in a new taxon described below, *A. riparium* Pillon and Hequet sp. nov.

Measurements with an XRF spectrometer indicate that the nickel content in dried leaves exceeds 1000 μg·g^−1^ in at least some individuals of six species: *A. brevipetalum*, *A. ellipticum*, *A. grunowii*, *A. montanum*, *A. riparium* sp. nov. and *A. vernicosum* (Table 1). The range of values observed between theses six species are similar. The median value for all species is below 1000 μg·g^−1^ and the highest value, 6670 μg·g^−1^, is observed in *A. ellipticum*. The only exception is *A. nitidum*, where values were consistently below the level of detection. Values observed with MP-AES on five individuals of *Argophyllum riparium* sp. nov. confirmed that it is a nickel hyperaccumulator: 1498, 1520, 1678, 2111 and 2214 μg·g^−1^ nickel in dried leaves.

## 4. Discussion

Seven species of *Argophyllum*, all endemic, can be recognized in New Caledonia. Two of them are variable and appear as species complexes: *A. ellipticum* or *A. grunowii*. It is not clear if this complexity may be caused by, e.g., hybridization [34,35] or cryptic species [36,37].

The obscure taxonomy of the genus led to uncertainty and discrepancy regarding the number and identity of the nickel hyperaccumulating species in different publications: *A. grunowii* and *A. laxum* [25,26,38], *A. ellipticum* [28], *A. brevipetalum*, *A. grunowii*, *A. latifolium*, *A. laxum*, *A. montanum* and *A. vernicosum* [27]. Novel measurements associated with a new taxonomy indicate that the nickel content in dried leaves exceeds 1000 μg·g^−1^ in at least some specimens of six species that can, therefore, be considered as nickel hyperaccumulators [39]: *A. brevipetalum*, *A. ellipticum*, *A. grunowii*, *A. montanum*, *A. riparium* sp. nov. and *A. vernicosum*. These six species are ultramafic obligates, except *A. ellipticum*, which can also occur on other substrates. The seventh species of the genus in New Caledonia, *A. nitidum*, never occurs on ultramafic substrates and consistently has nickel content below the level of detection. The genus *Argophyllum* is therefore similar to *Geissois* Labill. [16] (p. 50) (Cunoniaceae): Out of the 13 species endemic to New Caledonia, seven of the eight species occurring on ultramafic substrates are nickel hyperaccumulators [40].

Although six species of *Argophyllum* qualify as nickel hyperaccumulators, their nickel content do not reach the high values observed in some other New Caledonian species [27]. The genus could nevertheless be used for phytoremediation, phytoextraction or ecological restoration, but its multiplication and cultivation has apparently not yet been tested in New Caledonia [41]. The cultivation of the Australian species is considered easy [42].

## 5. Taxonomic Section

- Argophyllum J. R. Forst. and G. Forst. [1] (p. 29). Type:—*Argophyllum nitidum* J. R. Forst. & G. Forst.

non *Argophilum* Blanco [43] (p. 186) = *Aglaia* Lour. [44] (p. 173)

- Sectional names

1. Sect. Argophyllum

sect. *Brachycalyx* Zemann [17] (p. 271) nom. inval.

2. Sect. *Dolichocalyx* Zemann [17] (p. 271). Type [17] (p. 289):—*Argophyllum laxum* Schltr.

Zemann [17] did not provide a formal Latin diagnosis for either of the two sections. Section *Brachycalyx* included species from Australia and New Caledonia: *Argophyllum ellipticum*, *A. lejourdani* F.Muell. [45] (p. 33), *A. nitidum*, *A. nullumense* Baker [46] (p. 439) and *A. cryptophlebum* Zemann [17] (p. 283), whereas section *Dolichocalyx* included only New Caledonian species: *A. montanum*, *A. schlechterianum*, *A. grunowii*, *A. laxum* and *A. latifolium*. Because it includes the type of the genus, section *Brachycalyx* is not a valid name [47] (article 22.2). Zemann distinguished the two sections based on the length of the petals: calyx much shorter than the corolla in section *Brachycalyx*, calyx half as long but generally equal to the corolla in sect. *Dolichocalyx*. These sections are not maintained. They may not be reciprocally monophyletic groups, or this would imply that Australian and New Caledonian *Argophyllum* are not monophyletic groups. Additionally, calyx length is a labile character, i.e., it is variable within *A. ellipticum*.

- Argophyllum brevipetalum Guillaumin [22] (p. 25). Type:—New Caledonia, cours moyen de la Tontouta, le long des rives, ± 50 m, 14 December 1940, *Virot 375* (holo-: P00537620!)

*Argophyllum brevipetalum* is very similar to *A. ellipticum,* with which it shares the combination of yellow petals and hairy reddish coriaceous leaves, with the entire margin and inflorescences covered with red hairs. However, it occurs at a much lower elevation and differs in its smaller proportions (smaller leaves, slender petiole and stem). Guillaumin [22] (p. 25) segregated *A. brevipetalum* from *A. ellipticum* because of its calyx that is as long as its corolla, but this character is variable within *A. ellipticum* and is therefore not a reliable character alone on which to separate them.

- Argophyllum ellipticum Labill. [16] (p. 39). Lectotype (designated here):—New Caledonia, *Labillardière s.n.* (P00537623!, iso-: P00537624!, P00537625!)

*Argophyllum ellipticum* var. *comptonii* Baker f. [48] (p. 299), syn. nov. Type:—New Caledonia, Tonine, 30 September 1914, *Compton 1938* (holo-: BM001254561!)

*Argophyllum ellipticum* var. *ovatum* Pamp. [49] (p. 81), syn. nov. Type:—New Caledonia, *Labillardière s.n.* (holo-: FI018226!)

*Argophyllum ellipticum* var. *rigidum* Däniker [50] (p. 160), syn. nov. Type:—New Caledonia, auf des sud ost crête des Mt Humboldt, 7 November 1924, *Däniker 547* (holo-Z000015794!, iso-: Z000015795!)

*Argophyllum rufum* Vieill. ex Zemann [17] (p. 282) nom. nud.

*Argophyllum obovatum* Brongn. & Gris ex Guillaumin [51] (p. 60) [52] (p. 134) nom. nud. *Argophyllum vernicosum* var. *obovatum* (Brongn. & Gris ex Guillaumin) Guillaumin [21] (p. 277) nom. nud.

*Argophyllum ellipticum* is characterized by the combination of yellow petals, sepals much shorter or as long as petals, hairy coriaceous leaves (generally red and obovate in the most typical forms) with the entire margin and inflorescences generally covered with red hairs. *Argophyllum ellipticum* is widespread across the main island of New Caledonia, at mid- to high elevation, generally on ultramafic substrates, but occasionally on other, mostly poor, soils. It is a variable taxon, more robust in its dimension than the rare low-elevation *A. brevipetalum*. Specimens collected in the northeast of the island on non-ultramafic substrates, including the type of the species and of var. *comptonii,* have particularly short sepals (three times shorter than petals) and inflorescences with a long basal peduncle ramifying very distally. Most material collected on ultramafic substrates from Mont Humboldt and Mont Kouakoué, including the type of *A. ellipticum* var. *rigidum,* have leaves that are often revolute and whitish underneath, slender sepals (almost as long as petals), and shorter inflorescence ramified nearer the base. In locations in between these, generally on ultramafic substrates, the material displays a range of intermediate characters, which make this taxon difficult to divide further.

The name *Argophyllum obovatum* was published without a description and the specimen cited by Guillaumin [51] (p. 60), *Balansa 1814* (P00537626!, P00537627!) belongs to *A. ellipticum*. *Argophyllum rufum* has never been published with a description and was treated by Zemann [17] as a synonym of *A. ellipticum*.

- Argophyllum grunowii Zahlbr. [53] (p. 278). Type:—New Caledonia Thio, auf serpentinbergen, September 1884, *Grunow s.n.* (holo-: W1887-0007474!, iso-: NSW923234!)

*Argophyllum acinetochromum* Guillaumin [22] (p. 24), **syn. nov.** Type:—New Caledonia, pentes sud du Mont Kaala, ± 500 m, 2 November 1943, *Virot 1320* (holo-: P00537619!; iso-: NOU008828!)

*Argophyllum amoenum* var. *ovatum* Vieill. ex Guillaumin [52] (p. 133) nom. nud.

*Argophyllum brevistylum* Guillaumin [22] (p. 25), **syn. nov.** Type:—New Caledonia, près du sommet sud du Mont Kaala, ± 1000 m, 2 November 1943, *Virot 1318* (holo-: P00537621!; iso-: A01154254!, P00537622!)

*Argophyllum latifolium* Vieill. ex Zemann [17] (p. 285), **syn. nov**. Lectotype (designated here):—New Caledonia, ad montes prope Wagap, 1861-1867, *Vieillard 2199* (P00537630!, iso-: P00537631!, P00537632!). Remaining syntypes:—New Caledonia Kanala, *Deplanche 61* (P00537634!, P00537635!, P00537636!)

*Argophyllum laxum* Schltr. [54] (p. 118), syn. nov. Lectotype (designated here):?New Caledonia, Auf den Bergen bei Paita, 400 m, 7 October 1902, *Schlechter 14962* (P00537637!, iso:- BR699808!, E00346922!, HBG515583!, K000739390!, W1907-0011817!). Remaining syntypes:—New Caledonia, Auf den Bergen am Ngoye, 150 m, 30 November 1902, *Schlechter 15149* (B100715954!, BR699807!, E00346923!, HBG515582!, P00537638!, K000739391!, W1097-0011659!)

*Argophyllum laxum* var. *subintegrifolium* Baker f. [48] (p. 300), syn. nov. Type:—New Caledonia, riv. Ngoye, 20 October 1914, *Compton 2095* (holo-: BM000600291!)

*Argophyllum schlechterianum* var. *vestitum* Baker f. [48] (p. 299), syn. nov. Type:—New Caledonia, Taom, 2 December 1914, *Compton 2298* (holo-: BM001254563!)

*Argophyllum splendens* Vieill. ex Guillaumin [52] (p. 134) nomen. nud.

*Argophyllum grunowii* is characterized by the combination of yellow petals, and leaves and inflorescences covered with greyish hairs. Plants from southwest New Caledonia (e.g., type of *A. laxum*) have large leaves and inflorescences much longer than the leaves. Plants from the eastern coast (e.g., type of *A. latifolium*) have large leaves but inflorescences shorter than the leaves. Plants from the northwest (e.g., the types of *A. acinetochromum* and *A. brevistylum*) have medium-sized leaves with a very dense indumentum underneath and inflorescences about the length of the leaves. In spite of these apparent geographical trends, there are many intermediate forms and variability found within populations. Although not entirely satisfactory, a broad concept is adopted here. *Argophyllum grunowii* is widespread across the main island of New Caledonia, at low to high elevation, only on ultramafic substrates.

The name *Argophyllym amoenum* (published as “*amœnum”*) apparently never appeared in print except in combination with var. *ovatum*. The name was published without a description and the single specimen cited, *Vieillard 2639* (P06309317!, P06309318!, P06309319!, P06309320!, P06309321!, P06309322!) belongs to *A. grunowii*. The collection number for the type collection of *Argophyllum laxum* var. *subintegrifolium* is 2095 on the herbarium label but was printed as 2905 in Rendle et al. [48]. The name *A. splendens* was published without a description and was treated by Guillaumin [52] as a synonym of *A. laxum*. Zemann [17] cited two collections for *A. latifolium*, *Vieillard 2199* and *Deplanche 61*; one sheet of *Vieillard 2199* is chosen here as a lectotype. Schlechter [54] cited two of his own collections for *A. laxum*: *Schlechter 14962* and *15149*. The first one (from Païta) is chosen over the second one (from Ngoye), as it was collected in the area where most of the typical forms have been collected.

- Argophyllum montanum Schlechter [54] (p. 118). Lectotype (designated here):—New Caledonia, Auf den Bergen bei Yaouhé, 700 m, 15 October 1902, *Schlechter 15032* (B100715955!; iso-:P00537639!, K000739388!)

*Argophyllum schlechterianum* Bonati & Petitmangin [55] (p. 650). Type:—New Caledonia, forêt du mont Dzumacs, 900 m, October 1906, *Franc 566* (holo-: Z00054678!)

*Argophyllum montanum* is characterized by the combination of yellow petals, glabrous leaves and inflorescences covered by greyish hairs. *Argophyllum montanum* occurs in the southern part of New Caledonia, at low to mid elevation, only on ultramafic substrates.

The type specimen is unusual in having some toothed leaves. We follow Guillaumin [21] in treating *A. schlechterianum* as a synonym of *A. montanum*.

- Argophyllum nitidum J.R.Forst. and G.Forst. [1] (p. 30). *Argophyllum nitidum* var. *nitidum* J.R.Forst. & G.Forst. Lectotype (designated here): New Caledonia, *Forster s.n.* (BM000600294!, iso-: BM000600292!, BM00600293!, K000739389! B100296104!, BW04962010!, W0017375!). See Nicolson and Fosberg [56] for a complete list of putative isolectotypes.

*Argophyllum sericeum* Poir. [57] (p. 449) nomen. nud.

This species here is considered as endemic to New Caledonia, although some of the Australian species have sometimes been treated as varieties of *A. nitidum*. It is the only species from New Caledonia with white petals. Its calyx is particularly short (<2 mm). Its leaves are more chartaceous with a finer silvery indumentum than other species. Its inflorescences are silvery too, sometimes tinted with red. *Argophyllum nitidum* is widespread across the main island of New Caledonia, from Mount Koghis to Balade, and it is most common in the northern part, on non-ultramafic substrates and at low- to mid-elevation.

- Argophyllum vernicosum Däniker [33] (p. 165). Type:—New Caledonia, im dichten hohen Gebüsch der Crêten in 600 m Meereshöhe am Mt Koghi, 1 February 1926, *Däniker 2725* (holo-: Z000015797!, iso-: Z000015798!, Z000015799!)

*Argophyllum ellipticum* var. *oblongifolium* Brongn. and Gris ex Guillaumin [52] (p. 133). nom. nud.

*Argophyllum vernicosum* is characterized by the combination of yellow petals, short calyx, glabrous shiny leaves and inflorescences covered with reddish hairs. *Argophyllum vernicosum* occurs in the southern part of New Caledonia, at mid- to high elevation, on ultramafic substrates.

The name *Argophyllum ellipticum* var. *oblongifolium* (originally “*oblongifolia*”) was published without a description; the single specimen cited, *Balansa 1816* (P0537640!), belongs to *A. vernicosum*, and we therefore follow Guillaumin [21] in considering *A. ellipticum* var. *oblongifolium* as a synonym of *A. vernicosum*.

**Figure 1:**
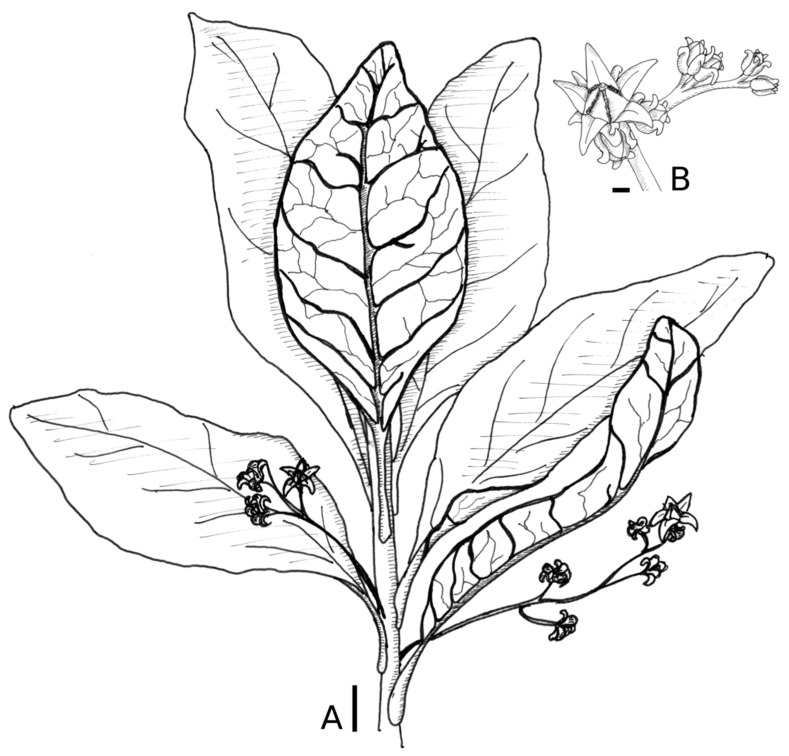
*Argophyllum riparium* Pillon and Hequet. (**A**) flowering branch, scale = 2 cm; (**B**) portion of an inflorescence, scale = 1 mm. Drawn from *Hequet 4662* by Thierry Sanchiz.

- Argophyllum riparium Pillon and Hequet, sp. nov (Figure 1)

*Diagnosis:* A species most similar to *Argophyllum vernicosum* Däniker, from which it differs by its larger leaves (generally ≥ 10 cm long), thicker twigs (at least 3–4 mm in diameter) and sparse branching. Its sepals are narrowly attenuated, nearly as long as petals, rather than shortly acute and not more than half the size of petals as in *A. vernicosum*.

*Type:* New Caledonia, Forêt de la rivière bleue près d’un creek, 23 April 1970, *Veillon 2137* (holo-: P04445141!, iso-: NOU024016 !).

*Description:* Shrub 1–2 m tall. Stipules absent. Hairs on new growth brown or rusty. Branchlet terete, thick, 3–4 mm in diameter at the insertion of inflorescences, reddish-brown. Leaves alternate, discolorous, at the end of the twigs. Petiole 14–41 mm × 2–2.5 mm. Leaf blade elliptic 9–18 cm × 2.5–6.5 cm, base acute, apex acute or retuse, 5–8 secondary veins on either side of midrib, margin entire to slight wavy, sometimes with a few teeth, few hairs invisible to the naked eyes (binocular × 4), glabrous and shiny on both sides. Inflorescence axillary, paniculate, densely reddish tomentose, 8–14 cm long, 15–60 flowers, primary axis 1/2–2/3 of the length of the inflorescence, secondary axis 10–50 mm long, branching spreading at 30–60° from adjacent branch. Bracts subtending primary ramifications of the inflorescence 4 × 0.5 mm, persistent. Floral bracts 2–3 mm, (?1–)2 per flower. Flower apparently bisexual, actinomorphic. Pedicels 2 mm long, flowering hypanthium cupular, 2.5 mm diameter. Calyx lobes 2.5 mm long; petals 3 mm long, yellow, corolla appendage yellow, 1 mm long. Stamen antesepalous, staminal filament 0.8 mm long, anthers 0.4 mm long. Style simple, 1.2 mm long, stigma swollen, entire, globose. Seed unknown.

**Figure 2:**
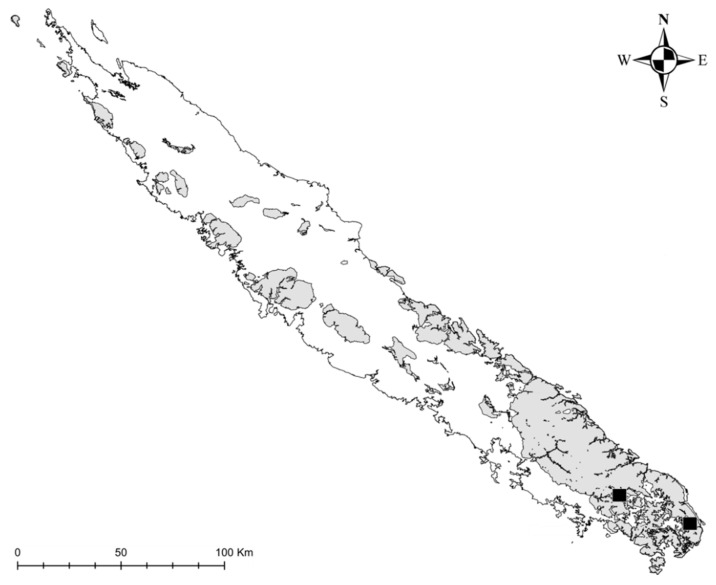
Distribution of *Argophyllum riparium* Pillon and Hequet (squares). Grey areas indicate ultramafic rocks.

*Ecology and conservation:* This species has been found in two locations (Figure 2). Most collections came from within the Rivière Bleue Provincial Park, where it grows near the rainforest edge on the banks of “rivière bleue”. All these collections may have been made from the same population. It appears in small, scattered groups of individuals, and its population size may be small. It was found recently in a second location in Plaine des lacs, growing along a stream close to the Kuebini river, 35 km apart from the type locality. Its ecosystem is undisturbed, and one of the two sites occurs within a protected area where the most likely disturbance might be floods, which can be severe during tropical storms. Its conservation status will be evaluated by the Plant Red List Authority–New Caledonia, and it could qualify as Vulnerable (VU D2).

*Paratypes:* New Caledonia, haute rivière bleue, 200m, 6 August 1951, *Baumann 15031* (P03609388!); ibid., 5 August 1951, *Baumann 15034* (P03609386!); Rivière Bleue S 22°5’-22°7’, E 166°37’–166°44’, 150 m, 1965, *Bernardi 9317* (P06234104!); Parc Provincial de la Rivière Bleue, S 22°05’47.4’’, E 166°38’16.4’’, 200 m, 8 November 2018, *Pillon & Isnard 1480* (P, NOU); Plaine des lacs: Kuebini S 22°13’9.89”, E 166°57’15.24”, 350 m, 23 September 2020, *Hequet 4668* (NOU107206!); Parc de la rivière bleue, S 22°05’49”, E 166°38’16”, 200 m, 18 March 2020, *Hequet* 4662 (NOU106963 !).

## 6. Identification Key to the Species of *Argophyllum* in New Caledonia

**1.** Leaf chartaceous, apex pointed, only primary and secondary veins distinct, leaf and inflorescences silky silvery (rarely tinted in red), petals white, calyx short (<2 mm), non-ultramafic substrates 
***A. nitidum***
**1’.** Leaf coriaceous, apex pointed or rounded, venation network distinct, leaves and inflorescences glabrous or covered with silver or red hairs, petals yellow, calyx short or as long as petals, generally on ultramafic substrate (occasionally on non-ultramafic but then on poor substrate for *A. ellipticum*)
**2**
**2.** Inflorescences covered with red hairs, leaf margin generally entire
**3**
**2’.** Inflorescences covered with white hairs, leaf margin entire to markedly toothed
**6**
**3.** Leaf blade covered with red hairs underneath, occasionally white when mature
**4**
**3’.** Leaf blade glabrous, shiny underneath
**5**
**4.** Overall plant slender, twig slender (diameter 2.5–4 mm), leaf 4–10 cm long, petiole 1.5 mm in diameter, petals and sepals about the same length, low elevation in Tontouta valley (<300 m) 
***A. brevipetalum***
**4’.** Plant robust, twigs thicker (diameter 4–6 mm), leaf 5–15 cm long, petiole 2 mm in diameter, sepals much shorter or as long as petals, widespread, mid- to high elevation (400–) 800–1600 m
***A. ellipticum***
**5.** Leaves small (mostly < 10 cm long), twig thin (c. 2 mm in diameter), sepals shortly acute, never more than half the size of petals, well-ramified shrub
***A. vernicosum***
**5’.** Leaves large (mostly > 10 cm long), twig thicker (3–4 mm in diameter), sepals narrowly attenuate, nearly as long as petals, sparsely branched shrub
***A. riparium***
**6.** Leaf glabrous, leaf margin entire, mostly south of Dzumacs-Yaté
***A. montanum***
**6’.** Leaf blade covered with pale to red hairs, leaf margin entire to toothed, widespread and variable 
***A. grunowii***


## Figures and Tables

**Table 1:** Nickel content (μg·g^−1^) measured from herbarium specimens with a handheld X-Ray Fluorescence (XRF) spectrometer. Values are in μg·g^−1^. LOD: Level of Detection. See Appendix A for individual values.

Species	Number of Specimens	Min (Ni)	Max (Ni)	Mean (Ni)	Median (Ni)
*A. brevipetalum*	4	<LOD	1675	483	129
*A. ellipticum*	12	<LOD	6670	1215	371
*A. grunowii*	40	<LOD	5694	1027	538
*A. montanum*	10	<LOD	1110	316	89
*A. nitidum*	9	<LOD	<LOD	<LOD	<LOD
*A. riparium*	4	173	1667	708	497
*A. vernicosum*	10	116	1872	608	387

## Appendix A

List of herbarium specimens analysed with an XRF spectrometer with herbarium barcodes and Ni content. Specimens with value above 1000 μg·g^−1^ are underlined.

***Argophyllum brevipetalum***: P03609387 (157 μg·g^−1^), P03609438 (101), P04351395 (<LOD), P06234113 (1676); ***A. ellipticum***: P00354794 (309), P03609379 (<LOD), P03609382 (2253), P03609437 (967), P03609439 (3223), P04284932 (608), P04445120 (118), P04445184 (6670), P04445187 (<LOD), P04445188 (<LOD), P06234110 (433), P06234114 (<LOD); ***A. grunowii***: P00217129 (167), P00217183 (1534), P00354673 (1351), P00537619 (207), P00537622 (3879), P00537637 (246), P00537638 (960), P03609286 (41), P03609287 (1581), P03609305 (737), P03609307 (485), P03609314 (320), P03609351 (437), P03609383 (2649), P03609384 (1238), P03609385 (5694), P04284517 (373), P04284521 (796), P04284933 (347), P04284938 (<LOD), P04284939 (489), P04351389 (587), P04351394 (1280), P04351397 (2514), P04351401 (752), P04351404 (782), P04445122 (127), P04445128 (461), P04445152 (229), P04445157 (73), P04445160 (<LOD), P04445164 (896), P04445165 (221), P04445166 (2778), P04445172 (672), P04445175 (1775), P04445179 (213), P04445180 (178), P06234106 (133), P06234119 (3896); ***A. montanum***: P03609289 (1110), P03609293 (1076), P03609294 (34), P04284918 (103), P04284920 (<LOD), P04351398 (511), P04445124 (33), P04445145 (50), P04445158 (165), P05563751 (75); ***A. nitidum***: P04284934 (<LOD), P04284935 (<LOD), P04351380 (<LOD), P04445131 (<LOD), P04445133 (<LOD), P04445173 (<LOD), P05563784 (<LOD), P05563794 (<LOD), P06234107 (<LOD); ***A. riparium***: P03609386 (1667), P03609388 (591), P04445141 (402), P06234104 (173); ***A. vernicosum***: P04284916 (132), P04284936 (402), P04351382 (345), P04445117 (1023), P04445162 (116), P05563809 (1872), P05563811 (217), P05563824 (387), P05563829 (977).
